# Prioritising Children and Young People with Disability in Research About Domestic and Family Violence: Methodological, Ethical and Pragmatic Reflections

**DOI:** 10.1007/s10896-023-00496-9

**Published:** 2023-01-28

**Authors:** Sally Robinson, Kristen Foley, Tim Moore, Kylie Valentine, Jala Burton, Amy Marshall, Melissa O’Donnell, Chris Brebner

**Affiliations:** 1 College of Nursing and Health Sciences, Flinders University, PO Box 2100, Adelaide, SA, 5001 South Australia; 2 Australian Catholic University, Melbourne, Australia; 3 University of NSW, Sydney, Australia; 4 Centre for Child Protection, University of South Australia, Adelaide, South Australia

**Keywords:** Intersectionality, Co-design, Contextual agency, Risk, Ethics, Participation, Research methods

## Abstract

**Purpose:**

The perspectives of children and young people with disability who experience domestic and family violence are under-researched, impeding the development of approaches that meet their needs. Knowledge gaps stem from the layered discursive positioning of disability, childhood/youth, or domestic and family violence in addition to the methodological, ethical and pragmatic complexity of research needed to understand their priorities and be attuned to their lived experience. This article explores methodological, ethical and practical challenges to centring their voices in research about domestic and family violence.

**Method:**

A conceptual framework of feminist disability theory and intersectionality informed our co-designed research, across three phases: (1) quantitative large-scale data linkage and case file analysis; (2) qualitative research with children and young people, their families and service providers and (3) stakeholder engagement workshops.

**Results:**

We reflect on how our research was able to prioritise the contextual agency of children and young people with disability, ways it could not, and other constraints.

**Conclusion:**

Children and young people with disability experiencing domestic and family violence hold an expert and unique vantage point on what happens to them. Amplifying their priorities for directing policy and organisational change requires more of researchers in terms of methods, but also more flexibility in how projects are funded to enable creativity and innovation. We call for collective attention to frameworks for supported decision-making and child ethics to progress inclusive research which recognises the importance of participation for children and young people with disability.

## Introduction

Children and young people with disability experience domestic and family violence at a higher rate than those of their peers who do not have disability (Jones, Bellis, Wood et al., 2012; Corr & Santos, 2017; Kyegombe et al., 2019). Children who have experienced violence are often treated as passive victims in policy, research and service settings (Øverlien, 2016), and are rarely engaged in research to understand their situations (Eliffe et al., 2020). This is exacerbated for children or young people with disability who are rendered as even more ‘vulnerable’, ‘at-risk’ or ‘incompetent’ (Brien, 2018); discursive positionings which isolates the individual from their context and reinforces the idea that they do not (or cannot) have agency or be meaningfully engaged without causing them harm (Abbas, 2017). While discussion about how to partner in research with children and young people has grown, this has not always translated to growth in methodological approaches that privilege the contextual agency of children and young people with disability, nor to those who experience domestic and family violence – let alone children and young people with disability who have experienced violence (Corr & Santos, 2017). In this article we focus on illuminating some of the methodological, ethical, and practical opportunities and challenges within such research.

### Purpose

The lack of knowledge about the perspectives and priorities of children and young people with disability who experience domestic and family violence reflects the lack of sustained focus on developing research approaches which accommodate diverse approaches to participation, rather than an inherent inability of children and young people to contribute (Danker et al., 2019; Rabiee et al., 2009). The moral imperative to allow children and young people with disability to speak for themselves rather than through proxies (Wickenden & Kembhavi-Tam, 2014) persists even if this means designing alternative methods to the most common qualitative and quantitative data collection practices. There have been important gains in exploring the family dynamics which (can) encase the participation of children in research (Dubois et al., 2021) as well as knowledge about how children and young people enact (and desire) agency during situations of violence (Katz, 2015; Callaghan et al., 2018, Noble-Carr, Moore & McArthur, 2020; Morris, Humphries & Hegarty, 2020; Arai, Shaw, Feder et al., 2021). Yet, research designs with children and young people at the intersection of both are less understood and broadly including a wider range of children in research about domestic and family violence is needed through adapted methods and approaches (Överlien, 2016; Franklin & Smeaton, 2017; Jones et al., 2017; Robinson & Graham, 2021).

The evidence we have around children and young people with disability that experience family violence is strikingly limited despite its prevalence (Robinson, valentine et al., 2020; Sutherland, 2021); possibly because it emerges from fragmentary service provision contexts (Lapshina & Stewart, 2019) complicated by historical legacies of segregation (Fawcett, 2016) and co-mingling ideologies of dis-ableism and vulnerability (Lid, 2015). Quite simply, the priorities of children and young people with disability experiencing abuse have rarely been sought via research (Jones et al., 2017), and what they have been allowed to contribute is constrained by logics of impairment and protection (Hollomotz, 2018). This gap in the literature is a failure of researchers and advocates rather than children and young people with disability to meaningfully contribute and communicate about their experiences (Raibee et al., 2009; Curran & Runswick-Cole, 2014).

This focus on the “inherent risks” associated with disability in children and young people has failed to alleviate or address their safety, while also positioning disability as a ‘risk factor’ for violence, rather than part of the context of children and young people’s social lives and worlds (Wayland et al., 2016). A focus on inherent risk also obscures their situational and environmental vulnerability (Hollomotz, 2011), that is, the multiple and interdependent contexts of children’s lives. Situational and environmental vulnerability are identified in a taxonomy of vulnerability by Mackenzie et al., (2014) as distinct from other forms of vulnerability in that they are context specific (Silvers, 2015, p.7). In contrast to inherent vulnerability, or that vulnerability which is shared by everyone and part of being corporeal, affective beings, situational vulnerability is formed by ‘personal, social, political, economic or environmental situations’. A focus on situational vulnerability instead prioritises the contributions and strengths of children and young people, their relationships, and the ways in which they navigate and counter violence and risk; while also contextualizing these in relation to underpinning cultural systems, values, expectations and histories (Njelesani, 2019).

In is in this context that our article describes the design and implementation of an intersectional, co-designed research project that aimed to centre the voices of children and young people with disability who experience domestic and family violence. Having elaborated the layered discursive positionings of childhood, disability, and violence, we now turn to the practical question of how to design and develop research projects that learn from these considerations and centre their voices. We demarcate three purposes for this article: first to describe our development of a research design that aimed to centre the voices of children and young people with disability who had experienced violence; next to discuss how and to what extent this design could be implemented; and finally to reflect on the limitations and propose future directions in this research area.

We recognise the paradox of focusing on our experience(s) as researchers while arguing to centre the priorities and perspectives of children and young people. Our article extends from the idea that researchers are both social subjects and social actors (Denzin, 2010) operating in specific social relations that influence knowledge production. Thus, in describing our research methodology, we hope to illuminate the ways in which we could reflexively centre children’s and young people’s voices during times of situational vulnerability, and the ways in which we could not, to draw attention to the ideas and practices which enable and constrict such methodologies. In this article, we elucidate how we integrated thinking about situational vulnerability, approaches to children’s rights and disability rights, and intersectional theory to form the project’s research methodology. A forthcoming article from the project focuses on the priorities of children and young people. Reports on the project findings are available in full and plain English versions through the Australian National Research Organisation for Women (ANROWS) website.

### Methodology

The *Connecting the Dots* project, commissioned by the Australian National Research Organisation for Women’s Safety, aimed to consider the various ways in which disability, age and gender intersect (along with other forms of difference), how this influences the ways children and young people with disability experience domestic and family violence, and the service responses they receive.

### Conceptual framework

The study was underpinned by feminist disability theory, a conceptual approach (Mays, 2006) which works to integrate analysis of gendered violence and the social model of disability. It emphasises the intersections of gendered and ableist norms that work to produce and reinforce the environments in which violence against disabled women occur; drawing attention to how services and responses can be effective and accessible. This conceptual approach aligns strongly with intersectionality theory, which articulates the ways that multiple forms of difference (including race, class, sexuality, age and disability) intersect to produce compounding disadvantages and exclusions (Shaw et al., 2012; Stubbs, 2015). Importantly, intersectionality and feminist theories both emphasise disability, gender, race and age are not simple differences between people but are axes of capitalist power and oppression (Crenshaw, 1991, 2017). In these circumstances, where institutions, services, policies, and workforces see and respond to one form of difference, people who occupy multiple oppressed categories experience layered and complex disadvantage (Stubbs, 2015). Using feminist disability and intersectionality theory demands methodological attention to the compounding intersections of disability, age and domestic and family violence, with consideration also given to Indigeneity, cultural and linguistic diversity, and rural/remote residence – both in supporting engagement with the research and in structuring research outcomes.

### Research Approach and Design

Children and young people with disability have a lot to say about what happens to them in situations of violence and risk when appropriate methods are used, particularly around what they need to be or become safe (Franklin & Smeaton, 2017; Hollomotz, 2018; Robinson et al., 2017; Jones et al., 2017). Consistent with this perspective, our study used a qualitative research design informed by descriptive phenomenology, leaving room for both a description of lived experience and understanding the meaning participants give to their lived experience (Van Manen, 2014; Corby et al., 2015). What appropriate methods might look like may vary in nearly every application to mirror the diversity of each child and young person, their disability/ies and communication preferences as well as the experience/s and impact/s of trauma. The use of concrete reference tools like picture cards and photo-stories (Hollomotz, 2018) as well as pictorial mapping can enable expression beyond words (Robinson et al., 2020; Robinson et al., 2018). Guided conversations using accessible language, like sign language (Jones et al., 2017) and plain English (Hollomotz, 2018) can support consent and discussion, as can trialling the methods with an advisory group (Franklin & Smeaton, 2017). Non-invasive interviewing (Robinson & Graham, 2021) can involve games and activities, where direct questions about trauma are not specifically asked – rather the child or young person is encouraged to share what is important to them. The opportunity for multiple meetings can increase access to participation and achieve more depth about a topic (Connors & Stalker, 2003) as can the presence of an adult who is familiar with the communication preferences of the person with disability, although this is complex in situations where that adult may also be involved or present during instances of violence (Jones et al., 2017).

Thus, an emerging body of literature shows that it is possible – and immensely valuable – to directly involve children and young people with disability in research about their experiences of violence. An undercurrent to this literature is the importance to glean as much relevant information about the contexts of their experiences which may decrease the burden on children and young people to participate, such as through literature reviews, surveys and interviews of service professionals (trauma, police, allied health) or policy actors, and parents (Franklin & Smeaton, 2017; Kyegombe et al., 2019) – or other avenues of relevant existing data. Principles of co-design, particularly advisory groups, can support decision-making about when and how to engage different stakeholder groups – important particularly in light of research showing that the (often unexplained) reluctance of well-meaning adults (including researchers, practitioners and policy-makers) to talk with children and young people about their experiences is interpreted as a failure to appreciate the impact of their abuse, to recognise their agency, and to value their views (Robinson & Graham, 2020; Moore et al., 2020).

Building on relationships with service providers to support recruitment and engagement in research may enhance participation (Martins & Sani, 2020); yet, they typically need encouragement to explore the potential risks and benefits of children’s participation about sensitive topics (Cater & Øverlien, 2014; Taplin, 2022). Yet, there are complexities to engaging proxies in understanding the perspectives and priorities of children and young people with disability about violence. Children (with and without disability) understand and experience the world in different ways to adults, and adult proxies cannot always provide a robust account of their experiences and views (Rogoff et al., 2018). This is particularly the case when experiencing family violence, during which children can hide their feelings and parents in ‘survival mode’ are focusing primarily on immediate safety needs rather than their children’s wishes (Noble-Carr et al., 2020). Despite attempts to shield them from violence, children experience direct and indirect victimization in ways under-appreciated by parents, researchers and service systems (Moore, Buchanan, Chung et al., 2020). Children are often agentic during periods of violence: regularly intervening, provoking, placating, protecting and helping their mothers and siblings (Morris et al., 2020; Noble‐Carr et al., 2020); yet they report feeling ‘invisible’ in unsafe environments (Eliffe et al., 2020; Moore et al., 2020) and this is problematically perpetuated by research that struggles to prioritise their perspectives about what happens to them.

Our study also attempted to use co-design methods. Co-design research is an increasingly popular methodological approach, with the primary intention to include relevant stakeholders meaningfully in design and implementation so that research processes and outcomes are relevant, valuable and acceptable to those who engage with them (Raman & French, 2022; Moll et al., 2020). Co-design approaches can improve the capacity of systems and services to meet diverse needs through partnership(s) with people typically marginalised and excluded; however, research may not be able to accommodate participants with intersecting social, health, and economic challenges (Mulvale, Mole, Miatello et al., 2019). Below, we describe how these theoretical and conceptual approaches influenced the development of the methodology.

### Research Methods

This research aimed to generate new knowledge about how children and young people with disability experience domestic and family violence – using mixed methods to bring together population level data and lived experience. The three-phase design connected findings from existing data sets to form a national picture of prevalence and risk, identifying data limitations and opportunities to improve policy and practice. It also prioritised the voice of children and young people with disability through qualitative and co-design activities that identified their support and service needs in the context of domestic and family violence, in addition to their priorities for safety and moving forward.

#### Phase 1

The first component of Phase 1 was the quantitative analysis of West Australian administrative linked data sets containing information about disability and/or domestic and family violence. We accessed several datasets (Police, Hospital Morbidity, Emergency Department, and Child Protection) and analysed the data for prevalence and risk. A second component of Phase 1 was a child protection case file analysis in the South Australian child protection jurisdiction to understand the nature and experience of domestic and family violence by children and young people with disability known to child protection services. The methods and findings of this phase are detailed in the companion report published by ANROWS.

#### Phase 2

Informed by emerging findings of phase one data analysis, this qualitative phase based on descriptive phenomenological inquiry aimed to explore needs and priorities of children and young people with disability and their families, as well as facilitators and barriers to violence-responsive services across a range of service sectors. Key questions were: what supports and services do children and young people with disability experiencing domestic and family violence need? What is important to them? What do they think? What are the hurdles and what is helpful in systems? Interviews and focus groups were conducted with each of the key participant groups:

- Children and young people with disability (aged 8 to 24 years) (n = 12): interviews focused on everyday life and priorities; support and service needs; priorities for generating safety; advice to other children and young people.
- Families/carers of children and young people with disability (n = 14): interviews focused on perspectives on support and service needs of children and young people; what helps their child prevent and mitigate effects of exposure to domestic and family violence.
- Practitioners across service types (n = 46): interviews and focus groups focused on opportunities and barriers to violence-responsive practice; system features to identify, prevent and mitigate effects of exposure to domestic and family violence for children and young people with disability.

The experiences of 36 children and young people are represented in our study. Twelve children and young people with a range of disabilities, aged 8–20 years, directly expressed their ideas and experiences during interviews. The experiences of a further 24 children and young people with disability aged between 6 and 24 years who were not able to participate directly for a range of reasons (including concerns about retraumatisation, children having complex support needs, and being absent due to child removal) are also represented in the report through the perspectives of their family members.

#### Phase 3

The final phase triangulated the results of the first two phases to build evidence for policy and practice. In workshops with a sub-set of participants, stakeholders and the advisory group (n = 22), we mapped out how to bring practice and policy into better alignment with the priorities of children and young people.

The research received ethical approval from Flinders University and three organisations involved in supporting fieldwork. Ethical approval for the use of data in phase one was provided through University of South Australia.

## Results

This section structures our reflections on how we were able to implement the research methodology (and ways in which we could not) according to the stages in which the research was conducted: (1) planning and funding; (2) co-design and co-production; (3) qualitative fieldwork processes; (4) analysis; and (5) dissemination.

### Planning and Funding

#### Mixed Methods to Support Children’s Participation

The first quantitative phase of the project brought together existing data sets. Overall, this supported a clearer understanding of existing gaps and helped us to understand the prevalence and risk of domestic and family violence for children and young people with disability at a population level. Bringing together population-level data, case-study analysis and qualitative research throughout the project was important in demonstrating the breadth and depth of this problem. Undertaking as much of the research as possible before engaging with participants also provided an evidence-informed foundation from which to start conversations with children and young people, families, and practitioners. It helped to minimise the burden to children so that they were not overtaxed during an interview (Devries, Naker, Monteath-van Dok et al., 2016).

#### Positioning Disability, Childhood and Violence

The discursive positionings (and intersections) of childhood, disability and domestic and family violence had to be addressed directly at the outset of our research, because Phase 1 and Phase 2 required differing conceptualisations of disability to guide data collection and analysis. Large scale data collection relies on diagnostic criteria and stable definitions across time and space, while social research privileges self-identification of lived experience and forms of difference. We used a social lens to enable our research processes to reflect and accommodate the subjectivity of impairment and the barriers people with disability face, which has been shown to improve the quality of inclusive research about domestic and family violence (van de Heijden, Harries, & Abraham, 2019). Age-related brackets of 7–12 and 13–18 are suggested useful for considering matters of research consent (Crane & Broome, 2017), but do not account for the intersectional considerations of children and young people with disability who may have also experienced trauma. To further translate our intersectional approach into a research method, we reasoned that young people with disability may not have the same social and developmental opportunities available to others, so broadened the age range in inclusion criteria. We actively resisted the idea that ‘truly voluntary’ consent (Cater & Øverlien, 2014) could exist, rather than attempting to evaluate the developmental stage of each child in the research to construct a valid ‘assent decision’. Instead, we sought informed consent that reflected the relational, emerging and interdependent nature(s) of children’s lives.

Children and young people and family members were not asked to provide information about their disability. Most children and young people in this study spoke about or were referred by family members as having cognitive disability – intellectual disability, autism, and developmental disability. Some young people spoke about their identity as disabled people, as Deaf and autistic people. Several people referred to the effects of living with multiple disability, including cerebral palsy, attention deficit hyperactivity disorder (ADHD), post-traumatic stress disorder, epilepsy, sensory impairments, particular syndromes, and mental health conditions including anxiety and depression.

We thought carefully about if/how we might conflate issues of risk, vulnerability and trauma, particularly for Aboriginal and Torres Strait Islander families and those for whom English is a second language. During the planning and funding phase, the general principles we had developed as a research team for applying intersectional theory provided practical guidance to critically engage with conceptualisations of risk and forms of difference during recruitment, data collection, analysis, as well as ethics applications and dissemination. Developing these ideas collaboratively amongst the multidisciplinary research team provided an early opportunity to elaborate the methodological rigour and theoretical coherence with the conceptual approach of intersectionality and situational vulnerability – while also ensuring shared understanding within the team about these links (Dubois et al., 2021). Our task in following these principles was complicated by challenges we encountered in recruiting, many of which are quite typical for qualitative research of this type. For example, we had included research funding for interviews in parts of rural and remote Australia, but it was not possible to travel due to COVID-19 lockdowns in the fieldwork period. The combination of geography, significant service dislocation and high rates of trauma experienced by children and young people with disability in the Aboriginal community we had planned to visit made fieldwork in that site unethical to implement with young people (van de Heijden et al., 2019).

#### Co-design and Co-Production

Consistent with the growing body of research pointing to the importance of involving co-researchers in data analysis and interpretation to centre their voices and perspectives, avoid tokenism and add rigour (Robinson, Fisher & Strike, 2020), we employed a co-researcher throughout the project. The aim of this role was to provide oversight, reflection and collaboration, and increase accessibility of processes and outputs. Author five was recruited to the project by recommendation from a collaborating organisation as a young person with lived experience of significant physical disability and experience in policy work. Her contribution to the project was important from the outset, but became deeper and richer as she grew into her role and gained experience and confidence. She provided advice about the broad scope and aims of the project, the accessibility of consent material and interview questions; read transcripts and coded data in Nvivo; took part in team meetings about emerging themes; reviewed reports; and developed the plain English report which had a dual benefit of synthesising the key messages. Jala particularly drew attention to the complexities involved for people with disability in accessing multiple services and dealing with various systems. Integral to making this role work well was ensuring accessible modes of work, including reduced and flexible working hours, and concentrated mentoring and supervision. Jala drew some very important and consistent threads through the project which drew new attention to the lived nature of disability and trauma, ensured that information was accessible, and ensured that we consistently recognised the research context as problematic, not children and young people.

### Qualitative Fieldwork

#### Reaching Children and Young People in the Research

We engaged with service providers to help recruit research participants on the logic that doing so would ensure that children and young people who were at heightened risk of experiencing distress were not invited into the study and those who were engaged were supported (van der Heijden et al., 2019). Service providers were asked to identify and contact families to participate. This often proved challenging. Domestic and family violence service providers struggled to engage with our research themselves, and to recruit families, due to structural pressures brought about by a large-scale funding restructure and the increased demand for services amidst the COVID-19 pandemic. While many expressed support for the research, we met a distinct reluctance from service providers to invite families, because they felt the topic was ‘risky’ for families who had experienced domestic and family violence, which may have highlighted the protectionist approach of some providers (Iacono & Murray, 2003). Partnering organisations linked the research team with families, namely parents. After talking with parents about the research (and most often, after mothers had participated in an interview themselves), we asked them to invite children and young people to participate. Some parents chose not to do this, believing that their children were too traumatised, or that their support needs would make their participation difficult. We do not know if the children and young people were involved in parents’ decision making.

To address recruitment shortfalls, we advertised the research on social media, resulting in direct contact by a small number of young people and family members. Those recruited via social media were less closely connected to services. Combining both referral pathways increased the diversity of the participant group and added an important intersection (i.e. service dis/engagement) to our research.

#### Accessible Materials and Processes

Prioritising accessibility for all participants drove the design of the recruitment and interview materials, with versions of each document created that used plain English and simple pictures as well as social stories, verbal information, and a consent script. Author five was a key contributor to these materials, helping to write and source appropriate pictures. The importance of accessible information was underscored by six of the 14 family members involved in our study self-identifying with disability, including cognitive disability. Translation into other languages, including Auslan, and live captioning were available for any participant who required it to improve accessibility. It was used in interviews, but logistics proved difficult for some of the workshops in phase 3, even with significant lead in periods. This constrained participation for people needing Deaf sign interpreting.

#### Ethics, Consent and Safety

Multiple ethical approvals were provided from universities and organisations involved in recruitment and the project wrestled throughout with several weighty ethical issues. A primary consideration in research with children about domestic and family violence is balancing the risk of protecting them from (further) trauma during research alongside making sure their voices are heard on issues that affect them (Goddard & Mundalay, 2009). Engaging participants in safe and ethical ways (recognizing that these words have contextual meanings) in partnership and negotiation with a series of “gatekeepers” (ethics committees, service providers and families) to facilitate the engagement of children is challenging (Martins & Sani, 2020). In our study, these stakeholders often perceived the balance of risk and harm differently to the research team and each other, given the sensitive topic (Taplin et al., 2022). They appeared to have adopted more risk-averse stances than the researchers or children and young people themselves (Cater & Øverlien, 2014), perhaps heightened by the focus in most family support services on adults – fewer services position children and young people squarely as the primary clients. Explicitly describing our research approach towards situational vulnerability and children’s contextual agency (Katz, 2019; Eliffe & Holt, 2019), then, was a critical step to building research partnerships that supported and nurtured children’s engagement (and consideration to their participation) throughout.

Families were central to this research project. Parents needed to have trust in the researchers and their ability to conduct the interviews safely and ethically, to consent to their children’s participation and to provide us with guidance about how to best understand what children and young people told us. In our research, we recognised family configurations would be nuanced by the communication and wellbeing needs children and young people with disability might have in a wider disability landscape that was fragmented and tumultuous (Yates & Dickinson, 2021). In line with recent calls for parents being fully engaged as research partners (Dubois et al., 2021), we spoke proactively with families about them and their children both being experts of their own situations, with children’s contributions being interdependent with their context, and articulated that their needs, preferences and priorities did not need to align during the research (Curran & Runswick Cole, 2014). We acted on the expert role of parents in asking for their guidance in appropriately communicating with children so that decision-making processes about research participation better reflected the context (Crane & Broome, 2017), and in using creative strategies that translated the participatory research design (Horgan, 2017) into the lives of children and young people with disability – because it engaged directly with how they communicate. Foregrounding and bringing the relationships of children and young people into this research helped to manage the sensitivity often associated with domestic and family violence research.

Creating accessible information helped in scaffolding consent processes that were centred on children and young people’s affirmative agreement to be involved. Where participants were under the age of 18, we asked family members to use these materials to help them talk about the project with their child or young person to gauge their interest and willingness to participate. We were keen to ensure that children and young people did not feel coerced into participation, given our primary recruitment was through families. We also spoke with the child or young person directly, using age-appropriate plain English, to explain who we were, why we wanted to communicate with them, what we would ask them, and what we would do with what we learned from them. We introduced ourselves to children and young people on separate occasions to the interviews, often when meeting with their parents, and gave them time to think about whether they would like us to come back and chat on another day. We also stated clearly that they were free to answer only the questions they wanted to. As per Silverman (2013), we used both verbal and written consent to prioritise the relationship and be flexible to children’s preferences, e.g. for reduced perceived formality and bureaucracy.

All children and young people and family participants were offered multi-store gift vouchers ($40AUD, the amount set by funding body and approved by ethics committees), to reflect the proportionate burden of their participation (van der Heijden et al., 2019). To limit the extent to which the reimbursement could be coercive, it was offered at the commencement of each interview, with assurance it wasn’t dependent on their participation thereafter nor to if/how they answered our questions. Information about the gift voucher was included in the documentation given to all participants before they consented to participate.

Guidelines for conducting research detail researchers’ ethical obligation to provide information about relevant services and supports to participants who may experience trauma as a result of their participation (World Health Organisation, 2005). Our research team believed that we also had an ethical responsibility to help families seek support if they continued to be experiencing violence or if the impacts of past violence were not being resolved (Berry, 2009). We prepared a flyer with the contact information of a wide variety of relevant services available across Australia, acknowledging that these are generally siloed into the domestic violence sector, the disability sector, and emergency supports. However, we were aware that children and young people with disability experiencing domestic and family violence experience many barriers and exclusions to services and supports, and were not always able to provide details of or referrals to services that would necessarily meet their (often complex) needs in a tailored way. It was confronting and difficult to be in a position where we could not offer practical support or access to services, knowing many of them were experiencing significant unmet need.

#### Approach to Interviews

Each participant had primary contact with the same researcher throughout their participation to support their sense of safety. Interviews with children and young people were undertaken either in person in their home (by preference) or via online video call. In both spaces, all children and most young people had their chosen family member with them or nearby. In negotiating when and where interviews were to be conducted, researchers assessed whether there may be any risks for participants or team members, including the risks of their confidentiality being compromised, of their safety being threatened in instances when a perpetrator of violence becomes aware of their participation, and of encountering a violent family member (Cater & Øverlien, 2014; Ellsberg & Heise, 2002).

Although the team was aware that there is little empirical evidence to suggest that being asked about traumatic events causes participants harm (Berry, 2009; De Prince & Freyd, 2006; Newman et al., 1997), researchers were proactive in monitoring family members and young people to ensure that they were comfortable in speaking about their experiences. Throughout the interview and in a post-interview follow up, participants were asked whether they had experienced any distress or had any issues that had emerged through their participation. None reported any distress caused by participation. Aligned with the aims of the research, which focused on the experiences and priorities associated with services during or after domestic and family violence, we did not ask children or their families about their experience of violence directly but allowed them to discuss their experiences unprompted.

The team recognised that effort was required to ensure that young people could participate meaningfully and that their individual communication and support needs were met. As the age range of participants ranged widely from eight to early-20s we tailored our approach to the varying developmental and linguistic capacities of participants when designing the interview questions and research tools (Dubois et al., 2021). We drew on a ‘toolkit’ of alternative tools and activities for communicating with children and young people (including drawing, mapping, picture cards and sensory activities), in recognition that communication does not always involve words (Rabiee et al., 2009). Pre-prepared social stories and activities were loosely structured around the research questions (e.g. collage activities that helped to explain what is important to you; Talking Mats cards which prompted discussion about things that help you feel happy and safe at home). These were available but not required to be used by children and young people. We encouraged children and young people to talk about what was important to them, by asking about or picking up on their interests and embodied expressions throughout the interviews. Interviews with children and young people were loosely structured, centred around their interests and relationships. This was also designed to reduce the potential of participants experiencing fatigue (van de Heijden et al., 2019). We designed the questions to be strengths-based, focusing on what was important to participants and what helps them the most. We used four main interview questions for children and young people (with some potential probing questions to explore their answers further if appropriate), so that they knew what to expect and to make it less intimidating:

- Can you tell me a bit about yourself and your family?
- What is important to you?
- What helps you and your family to be safe and happy?
- What do you think would help other kids who have the same kinds of experience as you to be safe and happy?

Questions were shared with many of the children and young people ahead of the interviews to reassure them about the focus of the conversation: that we would not be explicitly asking them about domestic and family violence and the questions were ones they could answer. Some children and young people said they liked knowing the questions ahead of time. Some children did talk about their concerns related to domestic and family violence, particularly those who felt confident in expressing themselves, once they felt comfortable with us.

#### Contextualising Voice, Meaning, and Understanding

**Fig. 1:**
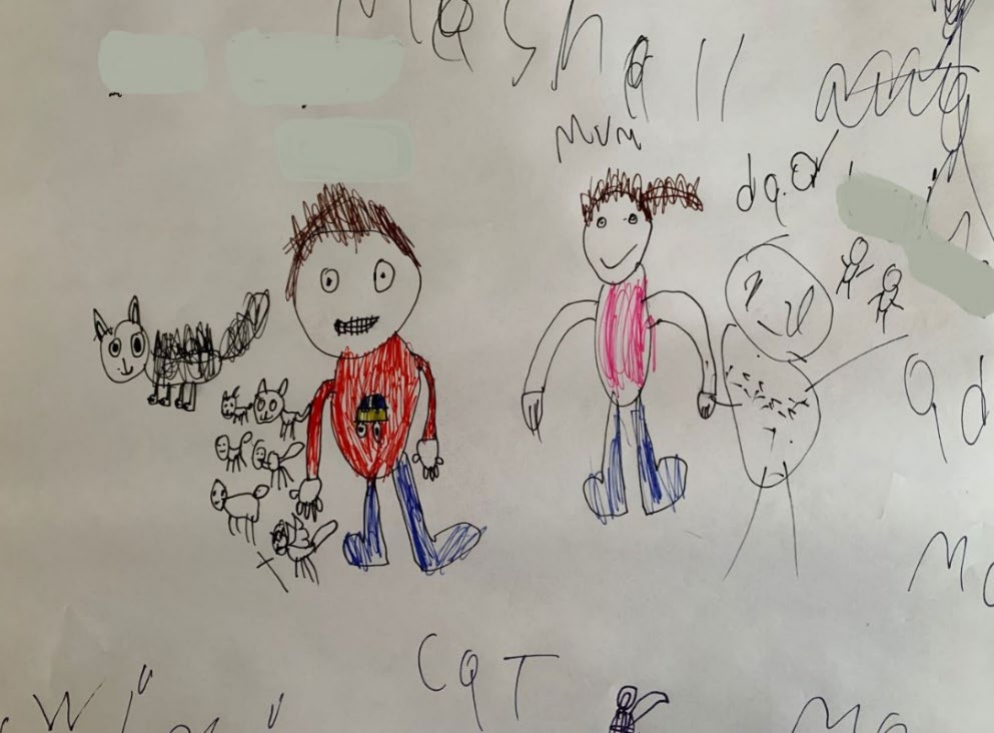
Isaac’s map

Pictorial mapping of children’s stories was used in interviews and appeared to be helpful in engaging children and young people and demonstrating that the researcher was listening to their stories. During interviews, children and young people were invited to draw images or write words or have researchers do this with them (Näykki & Järvelä, 2008). These maps highlighted the main themes and ideas that were shared (Fig. 1 provides an example). Researchers checked with participants that their pictorial summary represented what had been discussed, including at the end of interviews. This method has been shown to support people to engage with research findings in real time and accessibly review their ideas and correct any misunderstandings (Impellizzeri et al., 2017). We found this to be the case.

There were some moments during the interviews where it was difficult to understand what children and young people wanted to communicate. At some points, this was due to our lack of familiarity with their communication styles and the fact that we were not embedded in their lives. For example, Patrick and his family were provided with hotel-style crisis accommodation after they left the perpetrator, and housed in two separate rooms. Patrick had to go out into the main body of the hotel to reach his mother during the night and he and his siblings were disturbed by the lack of cleanliness of the accommodation and the pests, including rats, in the rooms. He drew a picture of one of these pests and himself on his pictorial map while we were speaking (refer to figure two), and told the researcher:

I was going to sleep, and I hear noise in the corner. I wake up mum, and I said ‘There’s something in my room! Some animal!’

It was only from the interview with Patrick’s mother that we discovered that at this time, he was sleeping in a separate hotel room in inappropriate crisis accommodation, and the whole family were distressed to be separated.

**Fig. 2:**
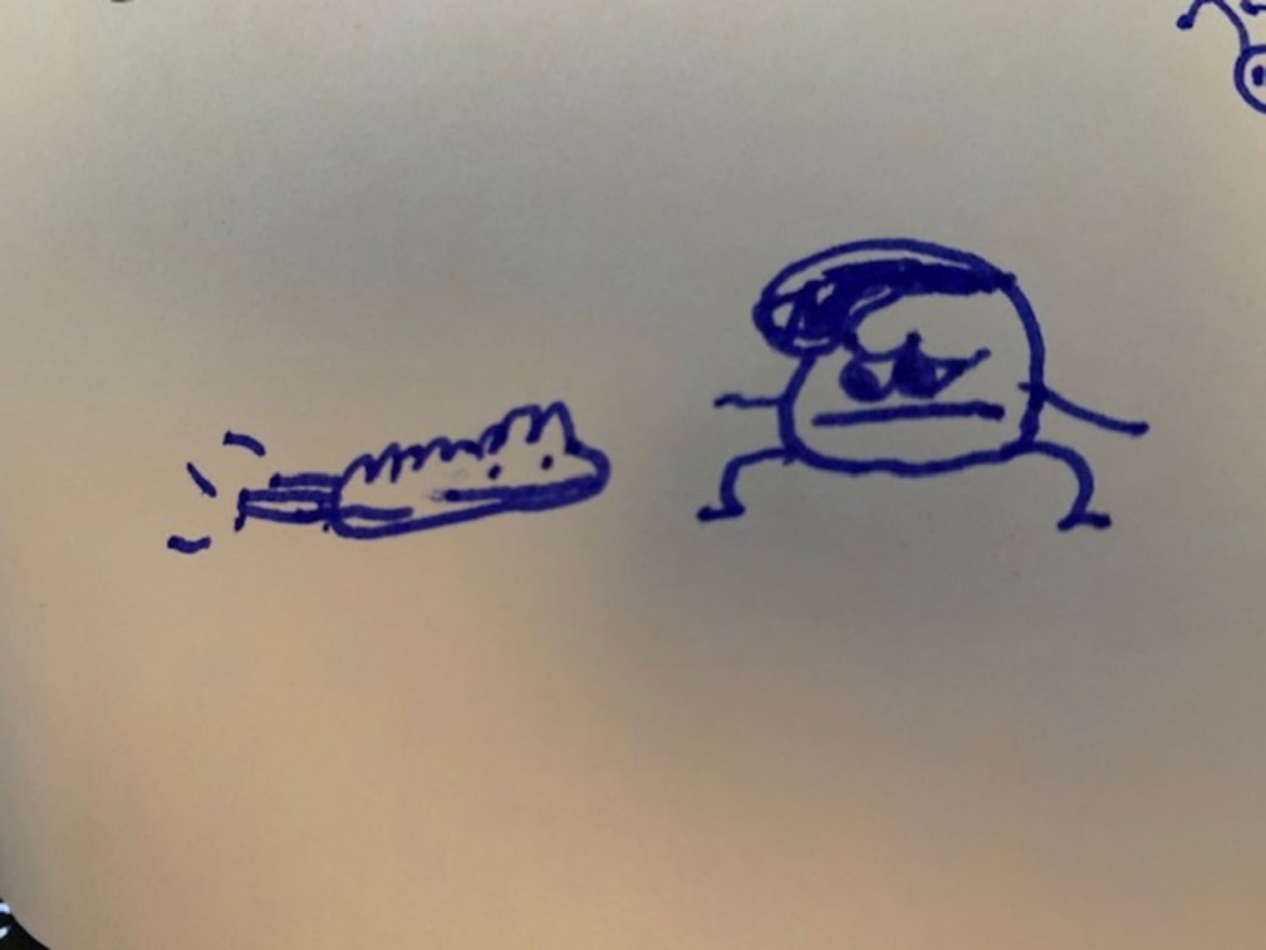
Patrick’s drawing of himself and the animal in his room

Although parents were often able to give us context and help explain what their children and young people had shared with us, we were cognisant of the fact that adults are not always in the best position to do this and that sometimes, particularly when they relate to children’s experiences of violence, adults’ accounts of how children understood their experiences and adults interpretations of children’s meanings do not represent children’s intent (Berry, 2009; Grover, 2004). As such, we spent time reflecting on both what we heard (from children and young people as well as their parents) and sought to make meaning of them both together and independently. Some of the time, our approach in interviewing parents before children provided important context that helped us to situate children and young people’s narratives. It was important not to assume we knew the answers to questions when we asked them of children, but it did help us to ask better questions of participants who had a concrete frame of reference. Knowing more about their preferences, strengths and experiences also supported this. Children and young people often provided thoughtful and insightful observations about their and their family’s experiences of violence. However, it was apparent that children and young people were not always afforded an opportunity to share their thoughts about what support would help them or their families.

It became apparent that some family members and practitioners held assumptions that children and young people did not have views about their experiences, or they were unable to share these views. Other parents and practitioners noted that this meant that children and young people were not always asked about their experiences or given opportunities to inform how they and their families are supported. As one participant noted:

It’s been almost impossible for them to really answer that question, because they’ve never had to or never had the opportunity to.

We are aware that assumptions like this about children’s ability to form and voice their views, from the “gatekeepers” involved in our research (parents and service providers), may have meant that some children and young people were excluded from our research.

### Data Analysis

We aimed to centre children and young people in the data analysis process by coding thematically against a framework built against the research questions.

The coding framework was confirmed by author one, four, and six, and reviewed by author five for inclusiveness and transparency. Pictorial data and researcher journal notes were also coded to include the non-verbal contributions made by children and young people in the research process. During coding, author one and four coded individually and after an initial meeting to refine the coding framework and process, met regularly to identify, consider and resolve divergent themes. Author five also read through qualitative transcripts and provided a key lens to help understand the priorities of our research participants. The thematic analysis followed Braun and Clarke’s six steps (Braun & Clarke, 2006). A high degree of consistency in themes across participant data and early coding saturation gave the research team confidence in the qualitative results. Following preliminary analysis by the research team, the initial results were presented in three online workshops to a mix of participants, stakeholders and advisory group members in phase three of the project and critically reviewed by the team. Discussing these helped us to identify priority areas which resonated with young people’s lived experience and areas of focus for policy change. Because of the comparative lack of power of children and young people’s perspectives in both research and policy, the team prioritised their perspectives in the analysis and writing. In practice, this meant analysing and writing up children and young people’s data holistically, before bringing it into interpretive dialogue with other stakeholders’ perspectives that were captured during data collection. This allowed us to build a richer picture of the shared priorities of children and young people with diverse experiences and backgrounds.

### Dissemination

The dissemination from our project was a significant undertaking and directed in part by the funding body, who have both expertise and trusted relationships in working with people who have disability. We worked with them to develop a knowledge translation and exchange plan from the inception of the project, which helped to frame multiple outputs for the involved stakeholders, including children and young people. There are few frameworks about how to disseminate research about gender-based violence in ways that suit people with people who require or prefer short and accessible summary material (van de Heijden et al., 2019). Author five’s work was critical here, to ensure that material was made easier to read and understand.

Significant limitations to the project outputs remain however, including funding limitations which precluded an accessible video version of the findings as well as outreach to other children and young people who may be interested in the research. The presence of family members in many of the children and young people’s interviews was both a strength and a limitation. They certainly provided clear support and encouragement for participation. While no family members appeared to constrain anything their children said, by being present, parents also cast a strong relational dynamic within which children’s family lives operated.

## Conclusion

Our research foregrounded the contextual agency of children and young people with disability in settings of domestic and family violence. We used an intersectional theoretical approach to develop a research methodology that aimed to create opportunities to centre the perspectives of children and young people. However, it is one thing to amplify voice and agency in methodology. Another is to respond – to really listen to children and young people with disability – in terms of harnessing priorities for policy and directing organisational change, so that they do not become ‘voices that fade away’ (Nortvedt et al., 2022, p.1467).

The children and young people who were involved in this study, and other similar research (Evang & Øverlien, 2015; Franklin & Smeaton, 2017), demonstrate that it is possible for children and young people with disability, including people with significant support needs, to contribute with meaning and depth in research of this sensitive nature. Children who have experienced challenges (including those with disability) may be experiencing high levels of situational vulnerability but this does not necessarily render them (more) vulnerable in a research context. There is no evidence that young people who have experienced challenges are more likely than their peers to experience distress within the research context; in fact, the opposite may be true when research is conducted appropriately (Berry, 2009; DePrince & Freyd, 2006; Newman et al., 1997). However, this is not without complexity: method and design *must* align with the realities of children and young people’s situational contexts.

Some children and young people who took part in our study chose to directly share with us their experiences of domestic and family violence and its impact on their lives. Some had things to say about their everyday lived realities that helped us learn from this everyday experience ways to support children and young people with disability who have experienced domestic and family violence. The perspectives of a larger group were provided to us despite their not being able to directly participate due to concerns about the impact of the level of trauma they had experienced, because they were in the child protection system, and/or because their parents deemed that their complex support needs would make it difficult for them to be involved in interview-based research. The concerns of family members about their children’s participation appeared to be well-grounded. A combination of structural and personal factors constrained young people’s participation. The structural barriers discussed by families were entrenched, multi-systemic, and out of the power of children and young people and families to resolve – such as poverty, engagement with the child protection system, and school exclusion. This was particularly evident for Aboriginal children with disability who experience family violence, who also experienced additional geographic remoteness and racism. With further adaptation to methods, resources, time, and effort, we can do better to hear directly from more children and young people with disability. But we will never hear directly from all. So, how do we progress?

When children and young people have a very concrete frame of reference (for example, due to cognitive disability or neurodivergence), there is work for researchers in connecting the dots between the things that matter to them, their lived experience, and implications for change-making. There are very real risks of disconnection from children and young people’s own interests and priorities in the analysis process. This may be where methods matter. Accessible ways to check with children and young people that our understanding of their lives is correct at a granular level are essential. So too are ways to check that the understanding generated through analysis resonates with children and young people’s experience and priorities. Scaffolding participation into research projects at multiple levels involves children and young people throughout the research in different ways, such as through co-research positions, advisory roles, participation in research, consultation on emerging findings, and in dissemination and knowledge exchange. Checking iteratively with the children and young people who do participate about methods for safe and ethical participation, emerging results, and what they want to be done with research findings weaves these processes into the project fabric. These methods and processes take time and resources, but may provide a level of confidence that children and young people are being prioritised throughout the research process, and the results reflect their experiences and perspectives more strongly as a result.

The use of mixed methods to support children and young people’s participation in sensitive research is an approach worth considering further. The quantitative data in this study served as both an ethical safeguard for the qualitative research for young people as well as producing important findings (Harari & Lee, 2021). In building the evidence about prevalence and risk from secondary data, we were able to identify key concerns facing children and young people as a group, and did not need to ask them individually about these painful experiences. Instead, we could focus on the things that might encourage positive change. Over time in this research field we have seen incremental improvements from funding bodies and ethics committees in terms of expectations around co-design – such as increasing expectations of high quality easy English consent forms and active encouragement of participation of the groups who are the subject of the research in advisory roles. We hope this trend continues with more meaningful change, such as employment of co-researchers embedded into grant requirements and widespread involvement of co-researchers involved in assessment and review panels for research grants (Nortvedt et al., 2022).

Conceptual and practical investment in research also needs to be made, looking towards frameworks that offer deeper developmental opportunities in methods that engage children and young people currently not well involved in interview-based research. For example, supported decision making (Bigby, Douglas, Smith et al., 2021) and child ethics (Graham et al., 2015) are both topical frameworks with practice application. It is past time to invest focus, time, energy, resources, and collaboration in identifying safe and ethical ways to expand the possibilities for participation in sensitive research for children and young people with disability, particularly children and young people with cognitive disability. Supported decision-making and child ethics frameworks together might help because they can align what we need to consider regarding human rights, fairness and equity, principles to underpin quality, and practice that supports accessible research. Both centre children and young people while foregrounding the important roles that supporters and allies play in contextualising participation and providing ethical safeguards. They can be customised to suit different cultural contexts, especially important for Aboriginal communities where the structural effects of violence are pronounced, and demand carefully crafted and community-led responses. These are practical examples of ways that critical disability studies can be embodied for children and young people with disability (Curran & Runswick Cole, 2014).

Children and young people with disability experiencing domestic and family violence hold an expert and unique vantage point on what happens to them. Conducting research in ways that can open and hold a space within which children and their agency can be made visible, recognized and respected, allowing them to share this expert knowledge. We hope that by demonstrating that this is possible their voices may influence policy, practice and further research.
